# The learning curve associated with the implantation of the Nanostim leadless pacemaker

**DOI:** 10.1007/s10840-018-0438-8

**Published:** 2018-08-13

**Authors:** Fleur V.Y. Tjong, Niek E.G. Beurskens, Petr Neuzil, Pascal Defaye, Peter-Paul Delnoy, John Ip, Juan Jose Garcia Guerrero, Mayer Rashtian, Rajesh Banker, Vivek Reddy, Derek Exner, Johannes Sperzel, Reinoud E. Knops, on behalf of the Leadless II IDE and, Observational Study Investigators

**Affiliations:** 1 Department of Clinical and Experimental Cardiology, Amsterdam UMC, location AMC, University of Amsterdam, Meibergdreef 9, 1105 AZ Amsterdam, The Netherlands; 2 Na Homolce Hospital, Praha, Czech Republic; 3 CHRU Albert Michallon, Grenoble, France; 4 Isala, Zwolle, The Netherlands; 5 Sparrow Research Institute, Lansing, MI USA; 6 Hospital Universitario Infanta Cristina, Badajoz, Spain; 7 Huntington Memorial Hospital, Pasadena, CA USA; 8 Premier Cardiology, Inc., Newport Beach, CA USA; 9 Mount Sinai Hospital, New York, NY USA; 10 Foothills Medical Centre, Calgary, AB USA; 11 Kerckhoff-Klinik gGmbH, Bad Nauheim, Germany

**Keywords:** Leadless pacing, Pacemaker, Arrhythmia, Learning curve

## Abstract

**Purpose:**

Use of novel medical technologies, such as leadless pacemaker (LP) therapy, may be subjected to a learning curve effect. The objective of the current study was to assess the impact of operators’ experience on the occurrence of serious adverse device effects (SADE) and procedural efficiency.

**Methods:**

Patients implanted with a Nanostim LP (Abbott, USA) within two prospective studies (i.e., LEADLESS ll IDE and Leadless Observational Study) were assessed. Patients were categorized into quartiles based on operator experience. Learning curve analysis included the comparison of SADE rates at 30 days post-implant per quartile and between patients in quartile 4 (> 10 implants) and patients in quartiles 1 through 3 (1–10 implants). Procedural efficiency was assessed based on procedure duration and repositioning attempts.

**Results:**

Nanostim LP implant was performed in 1439 patients by 171 implanters at 60 centers in 10 countries. A total of 91 (6.4%) patients experienced a SADE in the first 30 days. SADE rates dropped from 7.4 to 4.5% (*p* = 0.038) after more than 10 implants per operator. Total procedure duration decreased from 30.9 ± 19.1 min in quartile 1 to 21.6 ± 13.2 min (*p* < 0.001) in quartile 4. The need for multiple repositionings during the LP procedure reduced in quartile 4 (14.8%), compared to quartiles 1 (26.8%; *p* < 0.001), 2 (26.6%; *p* < 0.001), and 3 (20.4%; *p* = 0.03).

**Conclusions:**

Learning curves exist for Nanostim LP implantation. Procedure efficiency improved with increased operator experience, according to a decrease in the incidence of SADE, procedure duration, and repositioning attempts.

**Electronic supplementary material:**

The online version of this article (10.1007/s10840-018-0438-8) contains supplementary material, which is available to authorized users.

## Background

Leadless pacemaker (LP) therapy was introduced to address the limitations of traditional transvenous implantable pacemakers (PM) [1]. The Nanostim LP system (Abbott, Chicago, IL, USA), introduced in 2012, has revolutionized the state of pacing therapy. Reported short-term complication rates of LP therapy have been comparable to traditional PM therapy but were different in nature [2]. However, when interpreting these results, the expected learning curve associated with the implantation of a novel device using a unique set of tools must be considered. Procedure-related complications, such as cardiac injury, potentially relates to the novelty of the leadless technology and operator experience. As has been the case with other technologies, one can expect that the outcome and efficacy will improve with time and clinical experience.

Previous studies reported quantifiable outcome and performance learning curves associated with the introduction of cardiac interventions, such as cardiac resynchronization therapy (CRT; [3]), subcutaneous defibrillators (S-ICD; [4]), and transcatheter aortic valve replacement (TAVR; [5, 6]). To date, the learning effect of the Nanostim LP is unknown yet is of paramount importance since it (1) aids to the knowledge of the number of implants that have to be performed before reaching an acceptable level of competence, (2) may enhance and inform the appropriate training strategy, (3) is essential for the comparison with traditional PM therapy, and (4) is crucial to reach valid conclusions on its safety and efficacy.

We therefore sought to describe the learning curve for individual Nanostim LP operators in relation to serious adverse device effects (SADE) within 30 days. In addition, we aimed to evaluate the impact of operators’ experience on procedural efficiency, according to procedure time and need for multiple repositioning attempts.

## Methods

### Study cohort

This analysis included patients who were implanted with a right ventricular active-fixation Nanostim LP within two multicenter clinical trials conducted in Europe, the USA, Canada, and Australia. Data were collected until March 16, 2017 for the Leadless Observational study (clinicaltrials.gov no. NCT02051972) and April 10, 2016 for the Leadless ll IDE study (clinicaltrials.gov no. NCT02030418; [1]). Enrollment in the Leadless Observational study was temporarily paused from April 18, 2014 to June 2, 2014 because of the occurrence of two fatal cardiac perforations. Patients implanted prior and post-pause were included in the analysis. The implant technique of the Nanostim LP has been described previously [7]. All implanting physicians followed a validated implant training program organized by the device manufacturer. Both studies conform to the ethical guidelines of the Declaration of Helsinki. Approval was obtained by each participating site’s Institutional Review Board.

### End points

End points in this analysis were (1) SADE up to 30 days post-implant procedure, (2) procedure duration, (3) number of device repositioning attempts, and (4) pacing thresholds at implant. SADE were defined as any undesirable effect related to the device or implant procedure that resulted in death, life threatening illness, prolongation of hospitalization, persistent or significant disability, or incapacity. Procedure duration was defined as the time from venous access to removal of the introducer sheath. Device repositioning attempts were defined as the number of times the LP was implanted into the endocardium after the initial implant. All complications were reported by the participating sites and monitored by the study organization and were adjudicated by the Clinical Events Committee of each study.

### Statistical analysis

The combined data from the two studies were included in the analyses. The baseline characteristics were reported descriptively by experience quartiles using the mean ± standard deviation with the numbers of patients for continuous variables and numbers with percentages for dichotomous or categorical variables, unless otherwise indicated. *P* values were computed for continuous variables using Kruskal-Wallis test with a non-normal distribution data and for categorical variables using chi-square test, or as appropriate. The number and rate of SADE up to 30 days post-implant were presented, and the Kaplan-Meier analyses and log rank test were used to assess event rates across groups.

The impact of individual implanter experience at the time of the implant on outcomes was analyzed. The total number of implants performed by each implanter was summarized and distributed equally in experience quartiles among all implanters. The ranking order of all implants per physician was determined by the implant date and time, and the patients were binned in quartiles based on this ranking number. The first quartile represents the initial experience of operators: the first two implants; the second quartile: the third to fifth implant; the third quartile: the sixth through tenth implant; and the fourth quartile represents operators with the most experience (i.e., more than 10 implants).

Univariable analyses were performed to investigate whether patient characteristics, pre-/post-pause status, or study indication was associated with the end points analyzed. Logistic regression analysis was performed for the complications outcome, and a general linear model was fit for the outcome procedure time. In the multivariable analyses, backward selection was used in model selection with a significance level for retention of 0.15. All statistical analyses were performed using SAS version 9.3 (SAS Institute, Cary, NC). *P* values < 0.05 were deemed statistically significant.

## Results

The pooled cohort consisted of 952 patients from the Leadless ll IDE Study and 487 patients from the Leadless Observational Study, resulting in a total of 1439 patients who underwent a Nanostim LP implant performed by 171 implanters at 60 centers in 10 countries. The median number of implants per operator was 5 (range 1 to 86). Table 1 demonstrates the baseline characteristics per quartile categorized on gaining implant experience.

**Table 1:** Baseline characteristics per experience quartile

Characteristics	Quartiles				*p* value*
	Q1 (1–2)	Q2 (3–5)	Q3 (6–10)	Q4 (> 10)	
Number of patients	317	325	311	486	n/a
Implanters	47	43	46	35	n/a
Demographics					
Age (years)	76.6 ± 11.3	75.6 ± 11.7	75.1 ± 12.9	74.9 ± 14.1	0.4145
Male	212 (66.9%)	203 (62.5%)	180 (57.9%)	305 (62.8%)	0.1422
BMI	28.5 ± 6.1	28.5 ± 7.9	27.7 ± 5.9	27.7 ± 6.0	0.0811
Pacemaker indications					
Chronic AF with 2nd- or 3rd-degree AV block	195 (61.5%)	176 (54.2%)	170 (54.7%)	217 (44.7%)	< 0.0001
Sinus rhythm with 2nd- or 3rd-degree AV block and a low level of physical activity or short expected life span	36 (11.4%)	48 (14.8%)	36 (11.6%)	91 (18.7%)	0.0091
Sinus bradycardia with infrequent pauses or unexplained syncope with EP findings	87 (27.4%)	101 (31.1%)	105 (33.8%)	180 (37.0%)	0.0345
Medical history					
Congestive heart failure	53 (16.7%)	43 (13.2%)	37 (11.9%)	58 (11.9%)	0.2088
Hypertension	254 (80.1%)	249 (76.6%)	221 (71.1%)	323 (66.5%)	< 0.0001
Diabetes	83 (26.2%)	77 (23.7%)	64 (20.6%)	116 (23.9%)	0.4292
Peripheral vascular disease	34 (15.1%)	27 (11.4%)	21 (9.3%)	32 (12.2%)	0.2859
Coronary artery disease	114 (36.0%)	105 (32.3%)	96 (30.9%)	132 (27.2%)	0.0643
Myocardial infarction	41 (12.9%)	40 (12.3%)	37 (11.9%)	57 (11.7%)	0.9621
Unstable angina	10 (3.2%)	9 (2.8%)	7 (2.3%)	14 (2.9%)	0.9177
Prior PTCA/stents/atherectomy	46 (14.5%)	39 (12.0%)	51 (16.4%)	62 (12.8%)	0.3567
Prior CABG	51 (16.1%)	45 (13.8%)	25 (8.0%)	49 (10.1%)	0.0058
Ablation	30 (9.5%)	27 (8.3%)	41 (13.2%)	50 (10.3%)	0.2177
Medication					
Anticoagulants	201 (63.4%)	209 (64.3%)	186 (59.8%)	235 (48.4%)	< 0.0001
Antiplatelets	131 (41.3%)	109 (33.5%)	117 (37.6%)	173 (35.6%)	0.1978

*BMI* body mass index, *AF* atrial fibrillation, *AV* atrioventricular, *EP* electrophysiology, *PTCA* percutaneous transluminal coronary angioplasty, *CABG* coronary artery bypass grafting, *n/a* not applicable

*p values for continuous variables are computed using Kruskal-Wallis test and for categorical variables using chi-square test

### Impact on serious adverse events

**Fig. 1:**
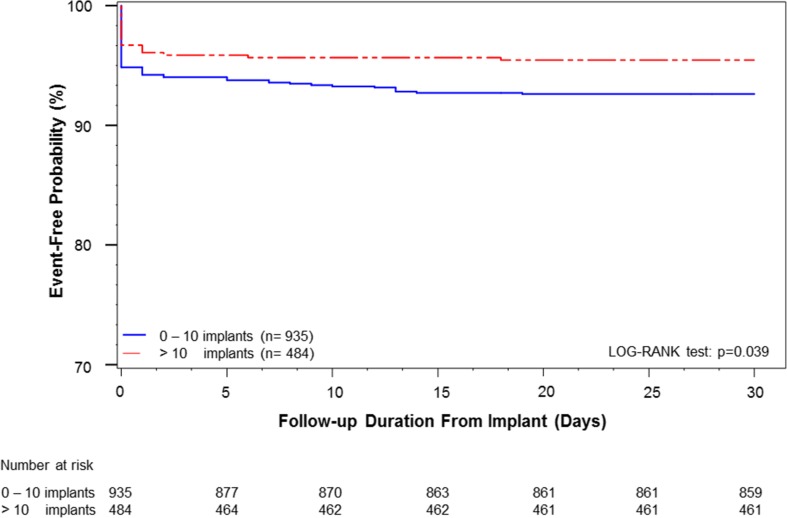
Kaplan-Meier curve illustrating the estimation of remaining free from SADE at 30 days post-implant. The red line represents patients who underwent a Nanostim LP implantation by physicians who performed more than 10 procedures (group 1). The blue line represents patients in whom the operator performed equal or less than 10 procedures (group 2). Thirty-day event-free rate following device implant in patients from group 1 was 95.5%, and patients from group 2 with 92.6% did not experience any type of SADE (log rank *p* = 0.039)

**Fig. 2:**
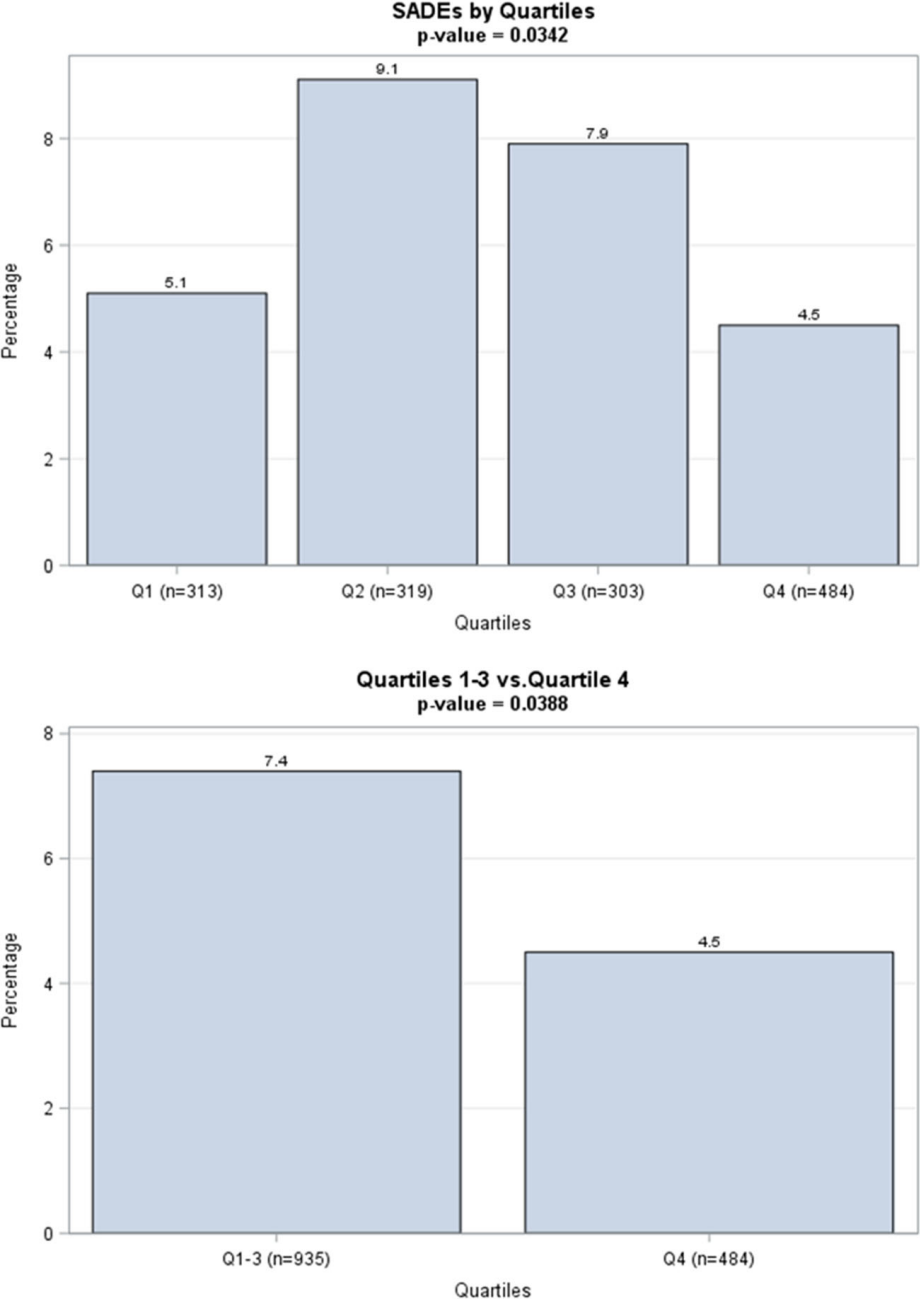
**a** Bar chart illustrating SADE in the first 30 days following Nanostim LP implantation per experience quartile. **b** Bar chart representing the SADE post-Nanostim LP implant within 30 days. SADE dropped significantly after 10 implants per operator (Q4 versus Q1 to Q3)

Of the 1439 included patients, 20 pre-CE mark patients with missing implant or SADE data were excluded, leaving a total of 1419 patients for this analysis. During a follow-up of 30 days, 91 (6.4%) patients experienced a total of 100 SADE, of whom 24 (1.7%) patients had a cardiac perforation, in 20 (1.5%) patients device dislodgement occurred, and 17 (1.2%) patients experienced vascular complications. Of the 24 cardiac perforations, 18 resulted in cardiac tamponade and 6 resulted in pericardial effusions without tamponade. In the six non-tamponade perforation cases, only two required intervention. There were two instances of cardiac perforations that lead to death of the patient. An overview of all SADE is illustrated in Table 2. In the multivariable logistic regression analysis, age (odds ratio [OR] 1.02; 95% confidence interval [CI] 1.001–1.004; *p* = 0.04), pre-pause indication (OR 2.72; 95%CI 1.15–6.41; *p* = 0.02), myocardial infarction (OR 2.02; 95%CI 1.17–3.47; *p* = 0.01), and non-right ventricular apex position of the device (OR 0.52; 95% CI 0.30–0.89; *p* = 0.02) were associated with the end point measure of SADE. The 4th quartile (i.e., > 10 implant attempts) was associated with a significant lower complication rate compared with the cumulative complication rate of the first three quartiles, 4.5 versus 7.4%, respectively (*p* = 0.038). The Kaplan-Meier curve showed that for implanting physicians who performed more than 10 procedures, 95.5% of patients remained free from SADE at 30 days post-implant, as illustrated in Fig. 1. Patients in whom the operator performed equal to or less than 10 procedures, the event-free rate of SADE was 92.6% at 30 days following LP implant (log rank *p* = 0.039). Cardiac perforation occurred in 2% of patients in quartiles 1 through 3 compared to 1% in quartile 4 (*p* = 0.197), as can be seen in the Supplementary File 1. In Fig. 2, the SADE rates per experience quartile are illustrated: quartile 1, 5.1%; quartile 2, 9.1%; quartile 3, 7.9%; quartile 4, 4.5%; and quartile 1 to 3, 7.4% versus quartile 4, 4.5% (*p* = 0.038).

**Table 2:** Serious adverse events in the first 30 days

Description	Number of subjects with events	Number of events	Percentage of subjects with events (*n* = 1419)
Cardiac perforation	24	24	1.7
Pericardial effusion without intervention	4	4	0.3
Pericardial effusion with intervention	2	2	0.1
Cardiac tamponade	18	18	1.3
Vascular complication	17	17	1.2
Access site bleeding event	7	7	0.5
AV fistula	4	4	0.3
Vascular access site: pseudoaneurysm	5	5	0.4
Perclose system malfunction requiring surgical intervention	1	1	0.1
Arrhythmia during device implantation	12	12	0.8
Asystole	2	2	0.1
Ventricular tachycardia or fibrillation	3	3	0.2
Conduction block	4	4	0.3
Other	3	3	0.2
Cardiopulmonary arrest	1	1	0.1
Device dislodgement	20	20	1.4
Device malfunction	7	7	0.5
Threshold elevation	5	5	0.4
Threshold elevation requiring retrieval of LP	1	1	0.1
Failure to capture/loss of capture	1	1	0.1
Thrombo-embolic event	6	6	0.4
Ischemic stroke	1	1	0.1
Probable pulmonary embolism	1	1	0.1
Thrombosis	1	1	0.1
Transient ischemic attack	3	3	0.2
Fever (unknown etiology)	1	1	0.1
Other	10	12	0.7
Total	91	100	6.4

*AV* atrioventricular, *LP* leadless pacemaker

### Impact on procedural efficiency

**Fig. 3:**
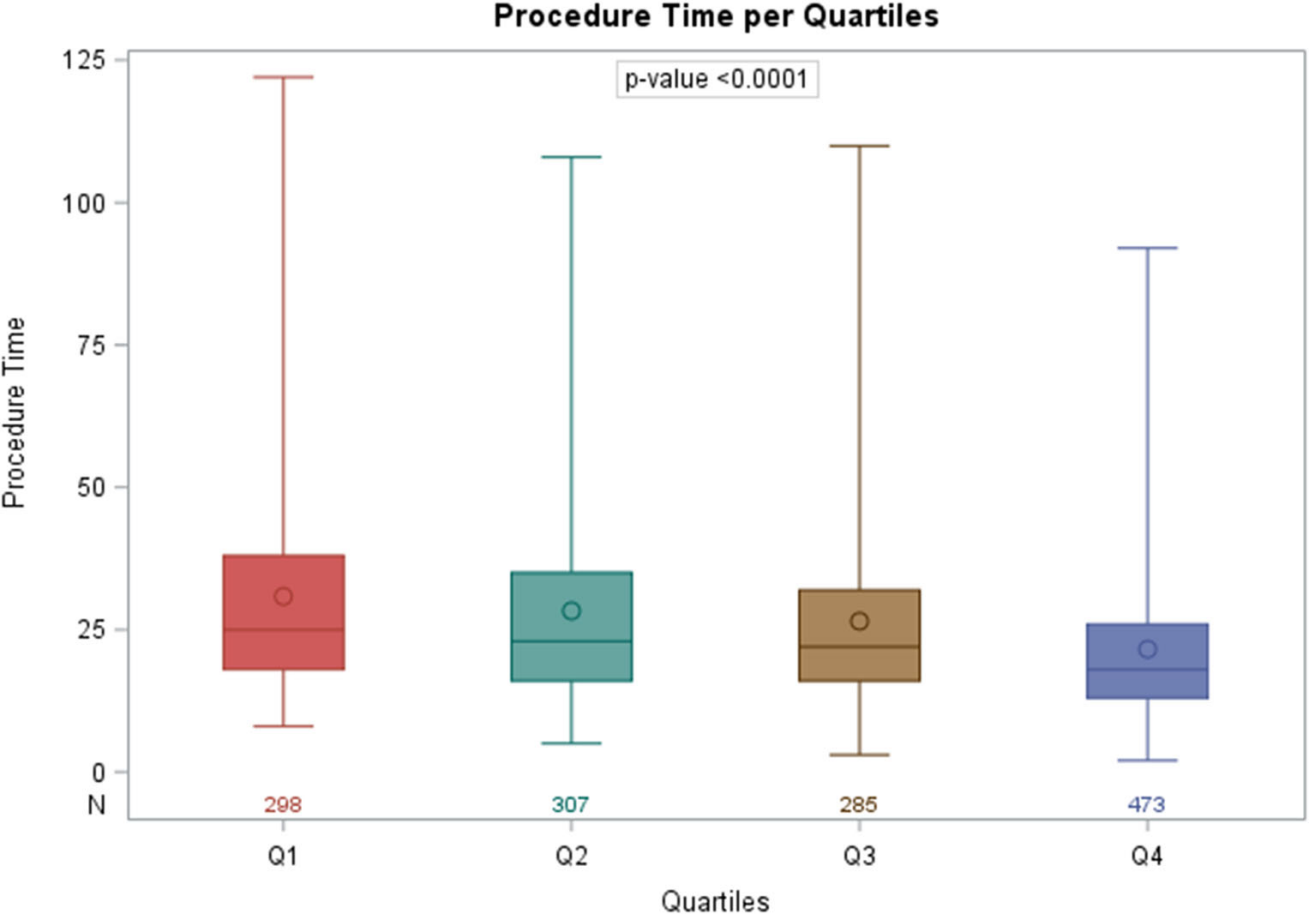
Boxplot showing Nanostim implantation time per experience quartile. The first quartile represents the initial experience of operators (1–2), the second quartile (3–5), the third quartile (6–10), and the fourth quartile represents operators with most experience (>10). The horizontal middle solid line of the boxplots corresponds to the median of the quartile. Total procedure duration significantly decreased across the procedure quartiles (*p* < 0.0001). N number of patients, Q quartile

**Fig. 4:**
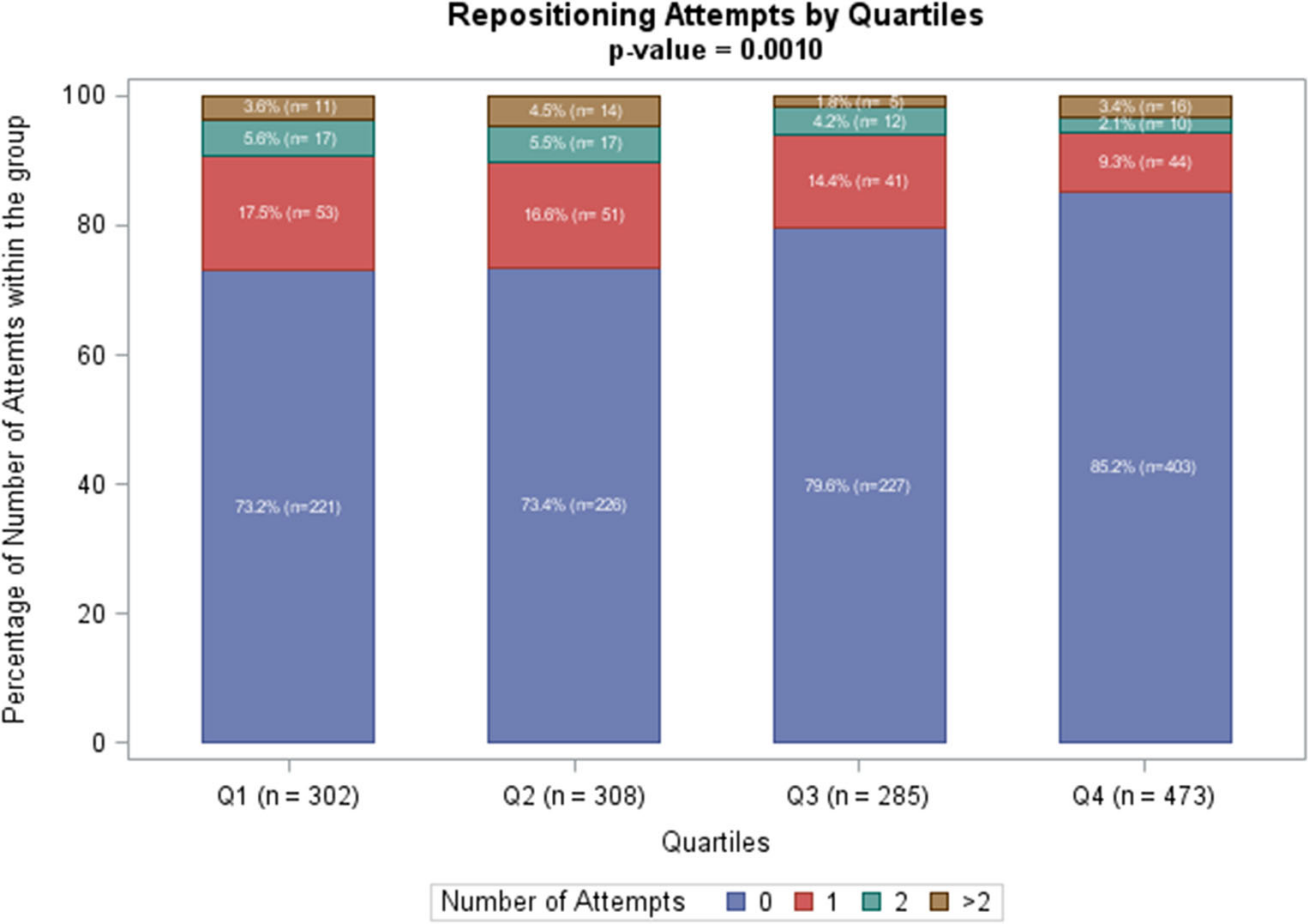
Bar chart illustrating the required number of device repositioning attempts per operators experience quartile. The purple area corresponds to no need for device repositioning, the red area represents one extra repositioning attempt, and the green and brown areas represent two and more than two additional repositioning attempts, respectively. The need for multiple repositioning attempts during the Nanostim implant procedure reduced with increasing quartiles (*p* < 0.001)

In 51 patients, the required data for procedural efficiency analysis was missing. These subjects were therefore excluded, resulting in a final cohort of 1368 patients. Total implant duration, which initially had a mean of 30.9 ± 19.1 min in the first quartile, decreased across the procedure quartiles to 21.6 ± 13.2 min (*p* < 0.001; Fig. 3). Overall, successful implantation within a single deployment of the device was achieved in 78.7%, which is seen in Fig. 4. Requirement for multiple repositionings during the LP procedure was significantly less common among operators with the most experience (14.8%), compared to quartile 1 (26.8%; *p* < 0.001), quartile 2 (26.6%; *p* < 0.001), and quartile 3 (20.4%; *p* = 0.03). Pacing thresholds at implant was not associated with operator experience (Supplementary File 2).

## Discussion

There are two principle findings of the current study. First, complication rates of Nanostim LP therapy were low throughout early implant experience but improved further after more than 10 implants per operator. Second, procedure efficiency significantly improved with gaining implant experience, based on a decrease in total procedure duration and reduction in the need for multiple repositionings.

Previous studies have shown that there is ample evidence for learning curves in newly introduced medical technologies, such as CRT, S-ICD, and TAVR [3–6]. These studies demonstrated consistent improvements in procedural parameters and metrics with increased experience until an asymptote was reached. Efficiency in performing CRT implants improved with increasing operator experience, with concomitant reduction of procedure and fluoroscopy time [3]. It took 10 implant attempts for the learning curve to reach its asymptote. For TAVR, 25 cases were needed before reaching an optimal level of competence, which translated in a decline of radiation and contrast exposure, together with a drop of complication rates [6]. Knops et al. demonstrated that complications following S-ICD implantation, which initially occurred in 9.8% of cases, significantly decreased to 5.4% over time and stabilized at an asymptote of 12 implant attempts per operator [4]. In the current study, the data also show a learning effect since operators in quartile 4 (most experience; more than 10 implants) had a significant lower complication rate compared with those who performed 1 through 10 procedures, 4.5 versus 7.4%, respectively. However, a different aspect of the learning curve was observed in our data. A notable low complication rate was seen during the initial experience of operators (i.e., 1 through 2 implants), followed by a significant rise (i.e., 3 through 10 implants) until a transition point was reached (i.e., more than 10 procedures) with concomitant lowest complication rates. This might partially be explained by the fact that all operators were tightly proctored during the initial implant attempts. In addition, one can imagine that the first and second implants of this novel technology were treated with the utmost care. A similar trend was observed for the occurrence of cardiac perforation during Nanostim LP implantation. SADE were associated with patient characteristics, such as older age and prior myocardial infarction, as well as procedural characteristics including right ventricular apex position of the device. In the initial phase of the LEADLESS Observational Trial, a right ventricular apical position was recommended; however, there were two instances of cardiac perforation that resulted in death which might partially be explained by the more easily penetrable right ventricular apex compared to the current recommended more apicoseptal positioning of the device. Enrollment in the Leadless Observational study was temporary suspended because of these fatal cardiac perforations. Subsequently, all operators were obligated to participate in enhanced training, involving extensive animal lab and video training. This is likely responsible for our finding that pre-pause patients were more prone to the development of SADE. Moreover, it acknowledges the impact of proper training and gaining experience on the performance learning curve of the Nanostim LP. Notably, there was a lower prevalence of chronic atrial fibrillation, hypertension, and anticoagulation use in the more experienced quartiles. This was balanced by more implantations in patients with sinus rhythm and infrequent pauses or syncope in the experienced quartiles compared to more indications of chronic atrial fibrillation with bradycardia in the less experienced quartiles. Quartile 4 may therefore reflect a healthier patient population which may be less prone to complications, such as significant pericardial effusion. In the later stages of the trial, it was generally more accepted to implant a single-chamber LP device in patients who had potential indications aside from chronic atrial fibrillation with bradycardia. In contrast, there were a lower number of patients with prior coronary artery bypass grafting in the higher quartiles, which might be expected to have the opposite effect as prior coronary artery bypass grafting might be protective against effusion.

The Micra Transcatheter Pacing System (TPS; Medtronic, Minneapolis, MN, USA) is the other clinically available LP for patients with a single-chamber pacing indication [8]. El-Chami and co-workers assessed the impact of operator experience on procedural outcomes with regard to the Micra TPS. They reported an overall 30-day complication rate of 2.9%. No significant association between operator’s implant number and complications on procedural quartile basis was observed. The complication rates among quartiles 1 to 3 (i.e., 1 through 12 implants) were 2.9 versus 2.7% in quartile 4 (i.e., more than 12 implants). There are differences between El-Chami et al. and the present study that merits emphasis. The Micra TPS study population contained 726 subjects, which is substantially lower than our 1419 cases. In addition, different cutoffs per procedural quartiles were used. Also, there are differences in the LP fixation mechanism and steerable catheter design which may contribute to the contradictory results [9, 10]. Moreover, the definition used for the primary safety outcome measure varies for the Nanostim LP and Micra TPS trials [1, 11]. The standard definition (ISO 14555 3.36) of SADE was applied in the Nanostim LP trial, whereas the Micra TPS trial established a more narrow definition (major complications) as the criteria for the primary outcome measure. Cardiac perforation by the active helix of the Nanostim is an uncommon phenomenon but is considered an important and potentially fatal complication. The incidence of cardiac perforation with the Nanostim LP was comparable to the rate associated with the Micra TPS and traditional PM [8, 12].

In line with El-Chami et al. results, procedure duration of the Nanostim LP implant significantly decreased by 30% over the experience quartiles [8]. Procedural experience may improve skill in the manipulation of the steerable catheter, which consequently results in a more efficient procedure over time. The Nanostim procedure duration observed in the fourth quartile is significantly shorter compared with the time needed to perform a conventional transvenous single-chamber PM implant (median 18 min versus median 39 min, respectively; *p* < 0.001) [13].

Our data showed that procedure experience impacts the number of device deployments required to obtain optimal pacing parameters. As expected, gaining experience enhances comfort with the steerable catheter which potentially abates the necessity for device repositioning. Of note, the need for multiple repositionings was low among all groups, and similar to El Chami et al. study, there was no significant association between procedure experience and the need for more than two repositionings [8]. As expected, pacing thresholds at implant were not associated with operator experience. This can be explained by the fact that pacing thresholds are affected by factors unrelated to operator experience such as the myocardial substrate, degree of injury at implant, and medications.

### Limitations

This large study is associated with several limitations. First, the study includes multiple centers and implanters which make it complicated to assess the learning curve per individual institution and implanter. Second, the learning curve data represents the experience accumulated before and after the pause of the Nanostim LP, which may be a confounding aspect in the analysis. Third, other potential confounders such as unrecorded comorbidities may influence the learning curve. Last, all operators involved in this study had experience in the usage of catheter-based procedures and may therefore be less representative of physicians without such experience.

## Conclusion

The incidence of SADE up to 30 days following Nanostim LP implant is significantly lower after 10 implants per operator. Performance efficacy improved over time, resulting in shorter procedure duration, and less frequent need for multiple repositionings. This indicates that the Nanostim LP implant procedure is subject to a learning effect. This knowledge has important implications with regard to physician education and training as well as when establishing competency requirements for implanting physicians.

## Electronic supplementary material

ESM 1 (DOCX 66 kb)

## References

[1] Reddy VY, Exner DV, Cantillon DJ, Doshi R, Bunch TJ, Tomassoni GF, Friedman PA, Estes NA, Ip J, Niazi I, Plunkitt K, Banker R, Porterfield J, Ip JE, Dukkipati SR, LEADLESS II Study Investigators (2015). Percutaneous implantation of an entirely intracardiac leadless pacemaker. N Engl J Med.

[2] Tjong FV, Reddy VY (2017). Permanent leadless cardiac pacemaker therapy: a comprehensive review. Circulation.

[3] León AR, Abraham WT, Curtis AB, Daubert JP, Fisher WG, Gurley J, Hayes DL, Lieberman R, Petersen-Stejskal S, Wheelan K; MIRACLE Study Program. Safety of transvenous cardiac resynchronization system implantation in patients with chronic heart failure: combined results of over 2,000 patients from a multicenter study program. J Am Coll Cardiol. 2005;46:2348–2356. 10.1016/j.jacc.2005.08.031 16360070

[4] Knops RE, Brouwer TF, Barr CS, Theuns DA, Boersma L, Weiss R, Neuzil P, Scholten M, Lambiase PD, Leon AR, Hood M, Jones PW, Wold N, Grace AA, Olde Nordkamp LR, Burke MC (2015). IDE and EFFORTLESS investigators. The learning curve associated with the introduction of the subcutaneous implantable defibrillator. Europace.

[5] Minha S, Waksman R, Satler LP, Torguson R, Alli O, Rihal CS, Mack M, Svensson LG, Rajeswaran J, Blackstone EH, Tuzcu EM, Thourani VH, Makkar R, Ehrlinger J, Lowry AM, Suri RM, Greason KL, Leon MB, Holmes DR, Pichard AD (2016). Learning curves for transfemoral transcatheter aortic valve replacement in the PARTNER-I trial: success and safety. Catheter Cardiovasc Interv.

[6] Alli O, Rihal CS, Suri RM, Greason KL, Waksman R, Minha S, Torguson R, Pichard AD, Mack M, Svensson LG, Rajeswaran J, Lowry AM, Ehrlinger J, Tuzcu EM, Thourani VH, Makkar R, Blackstone EH, Leon MB, Holmes D (2016). Learning curves for transfemoral transcatheter aortic valve replacement in the PARTNER-I trial: technical performance. Catheter Cardiovasc Interv.

[7] Reddy VY, Knops RE, Sperzel J, Miller MA, Petru J, Simon J, Sediva L, de Groot JR, Tjong FV, Jacobson P, Ostrosff A, Dukkipati SR, Koruth JS, Wilde AA, Kautzner J, Neuzil P (2014). Permanent leadless cardiac pacing: results of the LEADLESS trial. Circulation.

[8] El-Chami M, Kowal RC, Soejima K, Ritter P, Duray GZ, Neuzil P, Mont L, Kypta A, Sagi V, Hudnall JH, Stromberg K, Reynolds D (2017). Impact of operator experience and training strategy on procedural outcomes with leadless pacing: insights from the Micra Transcatheter Pacing Study. Pacing Clin Electrophysiol.

[9] St. Jude Medical. NanostimTM Leadless Pacemaker System. Executive summary for the circulatory system devices panel of the Medical Devices Advisory Committee. Version: 01/20/2016.

[10] Micra Transcatheter Pacing System (TPS). FDA panel pack for circulatory systems devices panel,February 2016

[11] Reynolds D, Duray GZ, Omar R, Soejima K, Neuzil P, Zhang S, Narasimhan C, Steinwender C, Brugada J, Lloyd M, Roberts PR, Sagi V, Hummel J, Bongiorni MG, Knops RE, Ellis CR, Gornick CC, Bernabei MA, Laager V, Stromberg K, Williams ER, Hudnall JH, Ritter P; Micra Transcatheter Pacing Study Group. A leadless intracardiac transcatheter pacing system. N Engl J Med 2016 Feb 11;374(6):533–541. 10.1056/NEJMoa1511643 26551877

[12] Mahapatra S, Bybee KA, Bunch TJ, Espinosa RE, Sinak LJ, McGoon MD, Hayes DL (2005). Incidence and predictors of cardiac perforation after permanent pacemaker placement. Heart Rhythm.

[13] Kirkfeldt R, Johansen JB, Nielsen JC. Conventional VVI pacing in Denmark. A benchmark for leadless pacing. EP Europace, Volume 18, Issue suppl_1, 1 June 2016, Pages i170.

