# Supplementary figures and images for: The learning curve associated with the implantation of the Nanostim leadless pacemaker
Source: J Interv Card Electrophysiol. 2018 Aug 13;53(2):239–47. doi: 10.1007/s10840-018-0438-8 (PMC6182715; doi:10.1007/s10840-018-0438-8)

**Supplementary File 1**


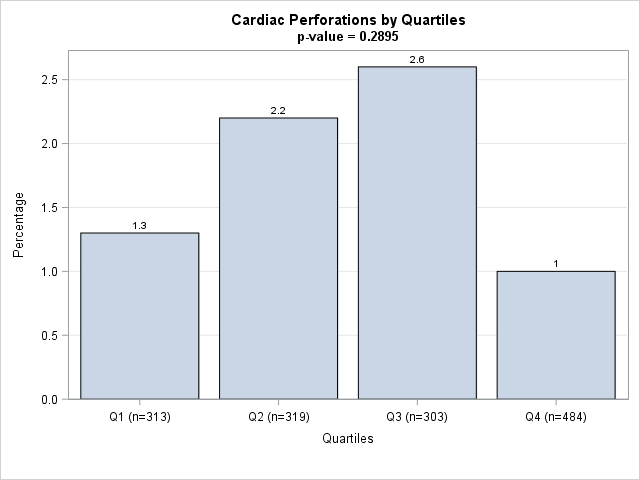

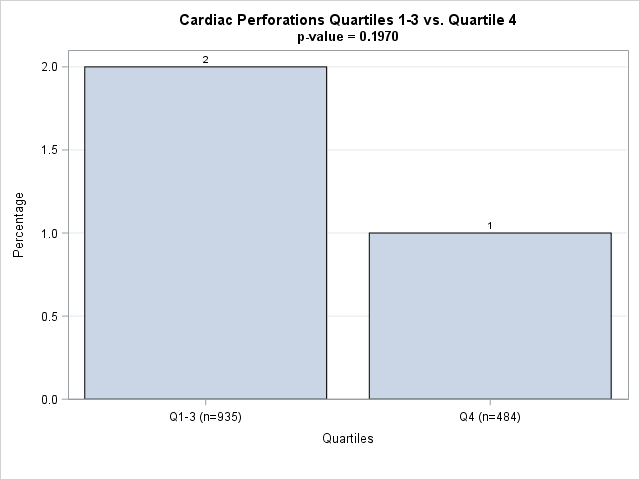


**A**

**B**

**Supplementary File 2**


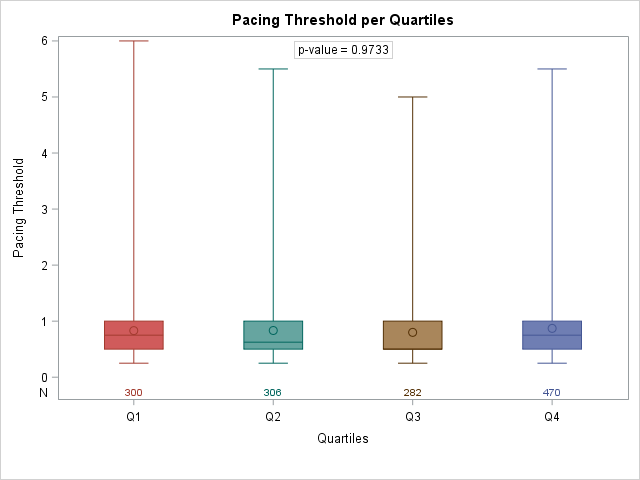

Supplement: Supplementary file 1 — (DOCX 66 kb) [file 10840_2018_438_MOESM1_ESM.docx]
